# The Impact of Home Interventions on Dry Eye Disease (DED) Symptoms and Signs in United States Veterans

**DOI:** 10.3390/ijerph22030438

**Published:** 2025-03-17

**Authors:** Drew C. Baeza, Johnathon Z. Penso, Dhariyat M. Menendez, Julio A. Contreras, Sarah Rock, Anat Galor, Naresh Kumar

**Affiliations:** 1 Bascom Palmer Eye Institute, University of Miami Health System, Miami, FL 33136, USA; db2612@mynsu.nova.edu; 2 Surgical Services, Miami Veterans Healthcare System, Miami, FL 33125, USA; 3 Dr. Kiran C Patel College of Allopathic Medicine, Nova Southeastern University, Fort Lauderdale, FL 33314, USA; 4 Department of Medicine, Miller School of Medicine, University of Miami, Miami, FL 33136, USA; jpenso@med.miami.edu; 5 Department of Health and Epidemiology, Miller School of Medicine, University of Miami, Miami, FL 33136, USA; dhariyat.menendez@gmail.com (D.M.M.); julio_con@yahoo.com (J.A.C.J.); srock@usc.edu (S.R.); nkumar@med.miami.edu (N.K.)

**Keywords:** dry eye disease, particulate matter, low-cost home interventions, osmolarity, Meibomian gland dysfunction

## Abstract

Background: The indoor environment can contribute to dry eye disease (DED) risk, but the effects of environmental modifications on disease are still uncertain. This study evaluated the effect of home interventions that modify the indoor environment on DED symptoms and sign severity. Methods: The prospective study consisted of two visits (6 ± 1 months apart). At each home visit, indoor environmental conditions (temperature, humidity, and airborne particulate matter) were monitored and at each clinical visit, DED symptoms and signs were examined. After the first visit, all participants received a report of their home air quality and 10 recommendations to improve their home environment. At the 6-month visit, participants indicated which interventions they implemented. Results: A total of 99 subjects participated in the clinical evaluation and home monitoring at baseline and six-month follow-up. Their mean age was 61 years, and 26% identified as Hispanic. Most had mild or greater DED symptoms (5-Item Dry Eye Questionnaire, DEQ5 ≥ 6), with an average DEQ5 score of 10.49 ± 5.51 at baseline. In total, 77% (n = 76) implemented ≥1 intervention with home ventilation (42.4%), air conditioner filter change (36.4%), and exhaust fan use (31.3%) being the most frequent. Overall, with every intervention implemented, tear osmolarity (change from baseline to 6 months) declined by 2% (log-transformed β = 0.02; 95% confidence interval (CI) = 0.00–0.03; *p* < 0.05), and Meibomian gland (MG) plugging declined by 14% (log-transformed β = 0.14; CI = 0.05–24; *p* < 0.05). Specific interventions had specific impacts on DED signs and symptoms. For example, osmolarity declined by a greater degree in those that implemented home ventilation, while DED symptoms improved to a greater degree in those that utilized indoor plants compared to those that did not implement these interventions. Conclusions: When provided with an objective report of home environmental conditions and remediation strategies, most participants voluntarily implemented low-cost home interventions, which reduced the severity of select DED symptoms and signs.

## 1. Introduction

Dry eye disease (DED) is an umbrella term defined as “a multi-factorial disease of the ocular surface characterized by a loss of homeostasis of the tear film, and accompanied by ocular symptoms, in which tear film instability and hyperosmolarity, ocular surface inflammation and damage, and neurosensory abnormalities play etiological roles” [1]. Many internal and external comorbidities have been associated with or have been found to contribute to DED such as age, autoimmune diseases, medications, and environmental exposures [2]. DED is a common condition in the general population and often presents to eye care practitioners [3]. Managing the various aspects of DED, on average, annually costs more than USD 11,000 per person in the United States [4].

Specific to environmental exposures, most of the literature has focused on outdoor environmental conditions, examining relationships between humidity, temperature, and air pollution (particulate matter (PM) (≤2.5 and/or ≤10 microns [PM_2.5_ and PM_10_, respectively]) and volatile organic compounds (VOC) such as nitrogen dioxide (NO_2_), carbon monoxide (CO), ozone (O_3_), and sulfur dioxide (SO_2_)) on various aspects of DED [5]. One retrospective study of United States (US) veterans captured DED health data using International Classification of Disease (ICD)-9 codes from the Veterans Administration database and meteorological data from the National Climatic Data Center and National Aeronautics and Space Administration [6]. Higher humidity was inversely associated with DED risk (Incidence Rate Ratio (IRR) = 0.927, 95% confidence interval (CI) = 0.926–0.927, *p* < 0.001) while temperature was positively associated with DED risk (IRR = 1.008, 95% CI = 1.007–1.010, *p* < 0.001) [6]. Other studies have focused on the role of PM in DED. A retrospective Chinese study examined 23,922 outpatient visits that occurred across 32 cities in relation to outdoor air pollutants (including PM_10_, PM_2.5_, NO_2_, CO, O_3_, and SO_2_) that were collected daily in each city. Individuals were defined as having a DED diagnosis using the Chinese dry-eye diagnostic criteria that included symptoms and signs of disease [7]. Exposure to high levels of PM_2.5_ (>81.21 μm/m^3^) for greater than 143 days a year (measured all year) was a risk factor for DED (Odds Ratio (OR) = 2.01, 95% CI = 1.79–2.26, *p* < 0.0001) [7]. Overall, numerous studies have demonstrated that extremes of temperature and humidity and high levels of outdoor air pollution have a detrimental effect on aspects of DED [8].

Fewer studies have examined the impact of the indoor environment on DED metrics. In a prior study, we interviewed individuals as they exited an older (n = 88) or newly constructed building (n = 102). The older building had higher levels of PM and microbial contamination compared to the newer building (24,436 particles vs. 12,213 particles ≥ 0.5 µm/feet^3^) [9]. Individuals leaving the older building reported a higher frequency of increased DED symptoms since entering the building compared to the newer building (37.5% vs. 28.4%) [9]. We also previously examined the impact of the home environment on DED measures in 97 veterans living in South Florida. In multivariable models, a unit increase in PM_2.5_ was correlated with a 1.59 increase in DED symptoms (Ocular Surface Disease Index (OSDI): 95% CI, 0.58–2.59; *p* = 0.002) and a 0.39 mm reduction in tear production (Schirmer: 95% CI, −0.75 to −0.03, *p* = 0.04) [10]. Our data suggest that similar to the outdoor environment, aspects of the indoor environment also contribute to aspects of DED.

There is a major gap in the literature on whether manipulating the indoor environment can mitigate DED symptoms and signs. However, the benefit of interventions that target the indoor environment has been investigated in diseases related to DED, such as asthma and COPD. In a randomized US clinical trial study of 391 patients with asthma, individuals were randomized to tailored educational interventions (in clinic or at home) versus no education [11]. Compared to the baseline, asthma control questionnaire score means improved to a greater degree in the intervention (baseline: 13.8 ± 3.9; 6 months: 16.2 ± 4.4, *p* < 0.001; higher value indicates better disease status) versus the no-intervention group, where scores were mostly stable (baseline: 14.3 ± 4.0; 6 months: 14.6 ± 4.9, *p* = 0.37) [11]. To address the gap in DED, in this prospective cohort study, we examined the impact of voluntary home environmental interventions on DED symptom and sign severity.

## 2. Materials and Methods

From 19 October 2017 to 30 August 2019, 99 participants were recruited from the Miami Veterans Affairs (VA) Healthcare eye clinic. Enrollment in the study required written consent and our inclusion criteria: central air conditioning (AC), living on or below the third floor, and being a permanent resident at the address until a follow-up visit in 6 months. Participants with any of the following health conditions were excluded from the study, as these variables could confound DED: contact lens use, ocular medications not including artificial tears, history of glaucoma or retinal surgery, or an active external ocular process. Participants were also excluded if they had human immunodeficiency virus (HIV), sarcoidosis, Sjögren’s disease, graft vs. host disease, or collagen vascular disease. The study was approved by the Miami VA and the University of Miami Institutional Review Boards (IRB approval #3011.05 and CR00012905, respectively). The study was conducted in accordance with the principles of the Declaration of Helsinki and complied with the requirements of the United States Health Insurance Portability and Accountability Act. Each subject had two paired clinical and home visits, approximately six months apart.

Questionnaires: All individuals filled out various questionnaires at their first clinical visit and during their 6-month follow-up. These captured demographic data (age, sex, race, ethnicity), past medical and ocular history, and medication use. Participants also filled out standardized DED symptom questionnaires, including the 5-Item Dry Eye Questionnaire (DEQ5) [12] and the OSDI [13]. The DEQ5 is a validated, five-item questionnaire that combines subject responses regarding “eye discomfort” (frequency and intensity), “eye dryness” (frequency and intensity), and “watery eyes” (frequency) during the past month. DEQ5 scores can range from 0 to 22, with higher scores indicating greater severity of symptoms. The OSDI is a 12-item questionnaire that assesses the frequency of dry eye symptoms and their impact on function on a 0 to 100 scale, with higher scores indicating greater severity of disease [13].

Ocular surface evaluation: All participants underwent a corneal sensation test via Belmonte aesthesiometer, as well as a standard ocular surface examination in both eyes, at their first clinic visit and as part of their 6-month follow-up. For corneal sensation, (1) detection and (2) pain thresholds in the right eye were tested (lower values indicate increased sensitivity) [14]. The ocular surface assessment consisted of the measurement of (3) tear osmolarity (TearLAB, San Diego, CA, USA); once in each eye; (4) eyelid laxity, sum of upper and lower laxity, each lid graded on a scale of 0 = no laxity to 2 = severe laxity, with a total possible score of 0–4; (5) conjunctivochalasis assessment in the medial, central, and temporal aspects of the lower eyelid, range 0–2 for each site, higher numbers indicate greater abnormality; (6) tear stability measured via tear breakup time (TBUT), 5 µL fluorescein placed, 3 measurements taken in each eye and averaged, lower levels indicate more tear instability; (7) corneal epithelial cell disruption measured by corneal fluorescein staining (CFS) to the National Eye Institute (NEI) scale, 5 areas of cornea assessed, range 0–3 in each section, total score range of 0–15; conjunctival epithelial cell disruption measured with Lissamine green (range 0–9), higher levels indicate greater epithelial disruption; (8) tear production measured with Schirmer’s strips (Tearflo, Hub Pharmaceuticals LLC, Scottsdale, AZ, USA) with anesthesia, lower levels indicate lower tear production; (9) inferior Meibomian gland (MG) plugging, range 0–3, and dropout, range 0–4, higher numbers indicate greater abnormality [15].

Home evaluation: Participants’ home environmental monitoring was conducted within 7 days following their clinical exam. This was done to avoid large variations in environmental conditions between the clinical and home visits. The monitoring took approximately 90 min. Air samplers and hygrometers were deployed in the room participants spent the greatest amount of time daily. We monitored humidity, temperature, and particle mass by two different sizes (≤2.5 µm and ≤10 µm in diameter, PM_2.5_ and PM_10_, respectively) using a portable particle counter (Aerocet 531; MetOne Instruments, Inc., Grants Pass, OR, USA). The device was stationed on a tripod stand approximately 1 m from the ground and 1.25 m away from the home’s central air conditioning unit or an air vent, whichever was in the room with the highest occupancy time. No cooking or other potential air pollution dispensing activities were allowed during the sampling period and the air handler (or AC) was running so that samplers could capture representative air of the indoor air circulating in the entire house. The particle counter was set to run 45 min on mass mode and 45 min on count mode. Mass mode estimated the mass of particles (micrograms per cubic meter of air) of varying sizes (1.0, 2.5, 7.0, and 10 μm). Count mode estimated the number of particles of two different sizes. In this study, PM_2.5_ and PM_10_ were the focus of interest, given prior data linking these known particle sizes to DED metrics [16].

Interventions: After sample collection, data were extracted and analyzed, and participants were provided with a report from their home monitoring, with levels and types of airborne particulate matter and pictures of their air samples. Educational guidance on how to improve their indoor environment, i.e., reduce air pollution and aeroallergens and improve thermal comfort, was also shared. This included ten recommendations based on the results of their environmental testing: (1) change central air conditioner filters every 2–3 months [17], (2) increase home ventilation by opening windows, especially after cooking and cleaning (depending on outdoor air quality) [18], (3) increase use of exhaust fan after use of stove/oven [19], (4) install high-efficiency air purifier to aeroallergens and particulate matter [20], (5) install plants in the home to reduce volatile organic compounds (VOCs) [21], (6) use vinegar and baking soda to remove for regular cleaning [22] instead of traditional bleach products [22], (7) keep indoor temperature below 72 degrees Fahrenheit [23], (8) keep humidity levels between 45 and 55% [24], (9) install ultraviolet (UV) light in central air conditioner system [25], and (10) remove carpet [26]. At the 6-month follow-up, individuals were asked which of the recommended interventions they implemented, and their responses were recorded in yes or no format for each intervention.

Statistical analysis: Descriptive statistics of home environment, DED symptoms and signs, comorbidities, and medicine use were assessed. Given correlations between some chronic conditions, we used factor analysis to group comorbidities and medications. Statistical analyses were conducted using data from the eye with the more abnormal DED metric. Participants’ changes in DED symptom scores (i.e., DEQ5 and OSDI) and signs were modeled with respect to the number of interventions and each intervention separately using linear or log-linear regressions depending on the distribution of outcome variables. Skewed variables were log(e) transformed to normalize them. All analyses were adjusted for demographics, comorbidities, prescription medication, and seasonality. While a *p*-value of <0.05 was considered significant, we also opted to give information for *p* < 0.1 to examine data trends. All analyses were conducted using STATA 14.2 (STATA Inc., College Station, TX, USA). *p*-values were two-sided. In our analyses, we opted to give information on all variables being compared as opposed to correcting the *p*-value (e.g., Bonferroni) since the latter methodology has its own limitations [27].

## 3. Results

A total of 99 veterans participated in both clinical and home monitoring visits at baseline and six-month follow-up. Their average age was 61 years, and most had other chronic diseases; approximately one-third of them were current smokers (Table 1). The most common comorbidities were depression (69.7%), arthritis (60.6%), and hypertension (60.6%). Baseline DED symptoms and signs and environmental metrics are presented in Table 2. Most individuals had mild or greater DED symptoms (DEQ5 ≥ 6, 92%), with an average DEQ5 score of 10.49 ± 5.5 (range 0–21) and an average OSDI score of 33.68 ± 25.3 (range 0–100) at baseline. DED signs varied widely with 8% of individuals having aqueous tear deficiency (Schirmer ≤ 5 mm) and 24% having tear instability (TBUT ≤ 5 s) in either eye.

### 3.1. Home Interventions and Change in Environmental Metrics

After conducting home monitoring, participants were provided with the report of their indoor air quality, which included information on temperature, humidity, and microscopic images of their air sample that showed sizes and shapes of airborne particles. They were also provided with guidance on how to improve their indoor air quality, which included 10 measures. Of the 99 participants, three-quarters (77%) voluntarily implemented one or more interventions (Table 3). Home ventilation (by opening doors and windows), air filter change, and use of an exhaust fan were the most frequently implemented interventions. Interestingly, DED symptom severity did not influence the implementation of interventions as a similar proportion of individuals with and without DEQ5 ≥ 7 adopted at least one intervention (*p* ~ 0.45).

Some environmental interventions had a beneficial impact on the home environment. In participants’ homes who changed their air filters, indoor PM_10_ concentrations declined to a greater degree than those who did not (1.24 µg/m^3^ vs. 0.164 µg/m^3^). A similar pattern was noted for PM_2.5_ concentration (1.31 µg/m^3^ vs. 0.97 µg/m^3^). Participants who installed an air purifier had a greater decrease in PM_10_ concentrations (1.34 µg/m^3^ vs. 1.19 µg/m^3^) but no difference in PM_2.5_ concentrations, as compared to those that did not install an air purifier.

### 3.2. Impact of Interventions on DED Symptoms and Signs

#### Number of Interventions

In the univariable analysis, participants who implemented at least one intervention saw significant improvements in tear osmolarity and inferior MG plugging scores as compared to those who did not use any interventions (Table 4). These findings held when considered in multivariable analyses, as with every intervention participants implemented, tear osmolarity from baseline to six months declined by 2% (β = 0.02; 95% confidence interval (CI) = (0.00–0.03); *p* < 0.05), the osmolarity difference between eyes declined by 28% (β = 0.28, CI = 0.01–0.45); *p* < 0.05), and Meibomian gland plugging declined by 14% (β = 0.14; CI = 0.05–24; *p* < 0.05) (Table 5, all values log-transformed).

### 3.3. Specific Interventions

All interventions except for air filter change and installation of UV light in the AC showed significant (*p* < 0.05) or a trend toward (*p* < 0.1) associations with select symptoms and signs of DED. Below, we focus our analyses on interventions that were implemented by at least 10% of the participants.

#### 3.3.1. Home Ventilation

Opening doors and windows for ventilation showed significant associations with several DED signs (Table 6). Participants who regularly opened their doors and windows for ventilation had a 3% greater decline in tear osmolarity (β = 0.03; CI = 0.00 to 0.07), a 72% greater decline in the osmolarity difference between eyes (β = 0.72; CI = 0.12–1.32), and a 32% (β = 0.32; CI = 0.08–0.55) greater decline in MG plugging than those that did not implement home ventilation (all values log transformed, *p* < 0.05 for all).

#### 3.3.2. Exhaust Fan Use

The use of an exhaust fan while cooking had an impact on DED symptoms and tear osmolarity. Participants who regularly used an exhaust fan had a greater decline in OSDI (33%) and osmolarity (3%) and a greater increase in tear production (21%) (all log transformed) compared to those that did not regularly use an exhaust fan (Table S1), values that trended toward significance.

#### 3.3.3. Vinegar and Baking Soda for Cleaning

Individuals who used versus did not use vinegar and baking soda for cleaning had an increase in conjunctival staining but a decrease in MG plugging, findings that trended toward significance (Table S2).

#### 3.3.4. Indoor Temperature Control

Keeping indoor temperature < 72 °F showed significant associations with the worsening of DED metrics (Table S3). Namely, participants who kept their temperature below 72 °F had greater increases in DEQ5 scores (β = 2.15; CI = −3.89–−0.41; *p* < 0.05) and MG dropout (β = −0.79; CI = −1.49–−0.08; *p* < 0.05, log-transformed) compared to those that did not keep their indoor temperature < 72 °F. Corneal staining also showed a greater increase in those that kept versus did not keep indoor temperature < 72 °F, but this finding did not reach statistical significance.

#### 3.3.5. Indoor Plants

Indoor plants showed significant associations with improvements in DED symptoms and MG plugging (Table S4). Participants who had indoor plants had a 52% greater decline in their OSDI score (β = 0.52; CI = 0.08–0.96; *p* < 0.05) and a 28% greater decline in MG plugging (β = 0.28; CI = 0.00–0.56; *p* < 0.05) compared to those that did not have plants in their home.

## 4. Discussion

In this study, we found that home environmental-monitoring-guided recommendations led a majority of participants to voluntarily implement interventions, which in turn improved a variety of DED symptoms and signs. Some interventions, such as home ventilation, AC air filter change, and exhaust fan use, were more frequently implemented than others, such as removing carpets or installing a UV filter in the AC unit, the latter interventions being more expensive to implement. Interestingly, frequently changing the air filter reduced coarse (PM_10_) but not fine (PM_2.5_) particle load, highlighting the limitation of this intervention in filtering fine particles and circulating gaseous pollutants. Moreover, the implementation of different interventions had different impacts on DED status, with the most consistent benefits seen with regard to tear osmolarity and MGD. Specifically, tear osmolarity and MGD metrics improved to a greater degree in those implementing versus not implementing home ventilation, while DED symptoms improved to a greater degree in those utilizing versus not utilizing indoor plants. These relationships remained significant when adjusting for demographics, comorbidities, and seasonality.

Our data build on prior studies that found relationships between adverse environmental conditions and DED. High and unstable tear osmolarity has been considered one hallmark of DED [10]. Adverse environments have been previously associated with tear osmolarity. A Brazilian study of 71 healthy taxi drivers and traffic controllers found that for each increase of 10 µg/m^3^ in PM_2.5_, a decrease of 10.9 mOsm/kg was noted in tear osmolarity (*p* < 0.05) [28]. This finding may reflect reflex tearing induced by air pollution. In our study, we found that opening doors and windows for ventilation, recommended to reduce inside particulate matter, correlated with reduced tear osmolarity.

Prior studies have also linked environmental metrics to MGD. One Chinese study recruited 864 individuals aged 20–80 years who spent 3–4 h outside and had no history of ocular surface abnormalities from five Chinese provinces across four regions (steel, coal, oil production, and densely populated for living, 2020 to 2023). Exposure was measured using the daily air quality index (AQI), PM_2.5_, PM_10_, O_3_, NO_2_, and SO_2_ from government air-quality monitoring sites in each province. Individuals were assigned exposure levels from 1 to 3 based on their average AQI exposure (level I: 0–50; level II: 51–100; level III: 101–150). Long-term exposure (defined as exposure over the prior month) to higher AQI levels was linked to more frequent and severe MG signs (loss, plugging, abnormal expression, and secretion) (all *p* < 0.0005) [29]. In our study, the environmental interventions most closely linked to MG improvements were home ventilation and the use of indoor plants.

While our study is unique in that it examined the impact of environmental interventions on DED, similar studies have been conducted in other disease states [30,31]. One Chinese study examined the use of a high-efficiency air purifier in 90 patients diagnosed with allergic rhinitis. Subjects were randomly assigned to an active or placebo air purifier which remained in their bedroom for >8 h per day. Individuals ran the air purifier for a 4-week period. At 4 weeks, the active air purifier group had a greater reduction in allergy symptoms (range 0 to 3 for a number of symptoms: congestion, sneezing, nasal itchiness, rhinorrhea, eye itchiness, ear/palate itchiness, eye redness, and tearing, total score 0–24) compared to the placebo group (mean difference = −9.0, *p* < 0.05) [31]. Similar findings have been noted in asthma. A US study of 274 low-income households with one child (age 4–12 years) with asthma assigned households to comprehensive (seven home visits, education, allergy control pillow, mattress encasements, and other allergy-free supplies) or minimal (single visit, limited resources) interventions. Symptom days decreased in both groups by a significant amount with the largest change being seen in the comprehensive group (4.7 days, 95% CI = 3.6–5.9 vs. 3.9 days, 95% CI = 2.6–5.2). The use of urgent health services bi-monthly saw a major decline in the comprehensive group (baseline 23.4% to 8.4%, *p* < 0.05) while no change was detected in the minimal intervention group [30]. While signs of DED were more robustly associated with environmental interventions in our study, indoor plant use was associated with significantly improved symptom scores in our population. These findings reinforce the concept that indoor environmental interventions should be considered, alongside medical therapy, in DED but further studies are needed in this regard.

As with all research, our study findings need to be considered in light of their limitations. First, environmental metrics do not exist in isolation and, in fact, interact with one another. For example, high humidity can increase airborne PM concentration [32], the time PM stays airborne, and its size [33]. In addition, high humidity can enhance the production and aerosolization of mold spores and endotoxins [34,35] (Investigating Homes for Mold, https://www.nbcmiami.com/news/local/investigating-homes-for-mold/47642/, accessed on 20 February 2025). This reality highlights the complex interactions between pollutants and the indoor microclimate that must be considered when making environmental recommendations. Second, our population consisted of United States veterans in a geographically restricted area, which limits generalizability. Third, we captured some home metrics, such as particulate air pollution but did not have information on other sources of air pollution, such as gaseous agents (e.g., O_3_ and NO_2_). Fourth, the implementation of guidelines varied by household, and multiple interventions were often implemented, limiting our ability to isolate the effect of any one intervention on DED metrics. Fifth, the unmasked and uncontrolled trial does not allow us to robustly assess whether improvements in DED metrics were directly related to the implementation of the intervention(s), especially as we captured home environment and clinic metrics over short time windows. However, it is reassuring that all subjects were treated similarly at baseline, as they were all provided with a report of their indoor air quality and guidance on different types of interventions, irrespective of DED status. Sixth, we cannot determine to what extent natural improvement or worsening of a participant’s condition impacted outcomes. Additionally, DED phenotypes varied, and not all individuals had abnormalities in all metrics.

Despite these limitations, this study supports the idea that the indoor air environment can impact ocular surface health. Interventions such as the ones we proposed, including improved ventilation and installation of high-efficiency particulate air filters, have been shown to decrease asthma symptoms and asthma-related hospital visits in prior studies [36]. When examining the implemented metrics, those most often adhered to were the ones with the least monetary cost or those requiring the least effort to implement. In our population, it was common to hear personal accounts of difficulty in following some recommendations, such as installing an air purifier or UV systems. This points to the need for future studies that more robustly investigate the effect of interventions on the indoor environment, including providing the recommended interventions and examining their relationship to improvements in DED symptoms and signs in DED-matched populations in intervention and control arms. With an increase in knowledge, data-driven policies, such as from the Environmental Protection Agency (EPA), can be put in place for recommendations and/or regulations of indoor air quality that may be beneficial for individuals living with DED and other chronic diseases such as asthma, chronic obstructive pulmonary disease, cardiovascular disease, and sleep disorder.

## 5. Conclusions

In conclusion, we found that most participants voluntarily implemented one or more interventions after receiving “objective” information on their home air quality. Implementing these low-cost interventions had beneficial effects on ocular surface health, namely with respect to tear osmolarity and MGD metrics. Our data provide support for considering environmental manipulations, along with medicinal therapies, as a way to decrease the burden of DED in the population. More studies are warranted to optimize recommendations, as they relate to specific DED sub-types.

## Figures and Tables

**Table 1:** Participants’ demographics, comorbidities, and medication use.

Demographics, %, (n) or Mean ± SD (n)	
Age (years)	60.9 ± 11.4 (99)
Sex, male	85.8% (85)
Race, White	46.5% (46)
Race, Black	53.5% (53)
Ethnicity, Hispanic	28.3% (28)
BMI (kg/m^2^)	32 ± 8.5 (99)
Smoking, current	28.3% (28)
Comorbidities, % (n)	
Hypertension	60.6% (60)
Hypercholesterolemia	48.5% (48)
PTSD	20.2% (20)
Depression	69.7% (69)
Arthritis	60.6% (60)
Sleep Apnea	39.4% (39)
BPH	21.2% (21)
Rosacea	1% (1)
Hepatitis C	14.1% (14)
Devices and Medications, % (n)	
CPAP	35.4% (35)
NSAIDs	26.3% (26)
ASA	36.4% (36)
Fish Oil	12.1% (12)
Multivitamins	61.6% (61)
Beta Blockers	12.2% (12)
Antidepressants	54.6% (54)
Antianxiety	52% (51)
Analgesics	56.6% (56)
Antihistamines	12.1% (12)

SD: standard deviation; BMI: body mass index; kg: kilograms; m: meters; PTSD: post-traumatic stress disorder, BPH: benign prostatic hyperplasia; CPAP: continuous positive airway pressure; NSAID: non-steroidal anti-inflammatory drugs; ASA: acetylsalicylic acid.

**Table 2:** DED symptom and sign scores at baseline.

Variable	Mean (SD) [Min–Max]
DED symptoms	
DEQ5 score (0–22)	10.49 (5.51) [0–21]
OSDI score (0–100)	33.68 (25.30) [0–100]
Corneal sensitivity	
Average detection threshold (mL/min)	79.44 (51.99) [15–410]
Average pain threshold (mL/min)	240.56 (133.54) [40–410]
Ocular surface signs ^β^	
Tear osmolarity (270–350 mOsm/L)	316.91 (19.06) [289–375]
Difference in osmolarity between eyes (mOsm/L)	15.24 (14.62) [0–80]
Sum score of eyelid laxity (0–4)	0.51 (0.81) [0–4]
Conjunctivochalasis scale (0–6)	1.15 (1.02) [0–5]
Corneal staining (0–15)	1.67 (2.13) [0–10]
Conjunctival staining (0–9)	0.58 (1.28) [0–8]
Tear breakup time (seconds)	8.09 (3.67) [3–25]
Schirmer wetting by tears (0–35 mm)	17.33 (8.88) [2–35]
Inferior Meibomian gland plugging (0–3)	1.45 (1.25) [0–3]
Inferior Meibomian gland dropout (0–4)	1.66 (1.11) [0–4]
Home environment	
Indoor dry bulb temperature (°C)	24.94 (2.84) [15–38]
Indoor relative humidity (%)	63.48 (20.59) [25–100]
Indoor heat stress index (°C)	25.66 (4.38) [14–46]
Indoor airborne PM_2.5_ concentration (µg/m^3^)	2.13 (3.66) [0–19]
Indoor airborne PM_10_ concentration (µg/m^3^)	6.69 (6.68) [0–30]

DED: dry eye disease; DEQ5: 5-Item Dry Eye Questionnaire; OSDI: Ocular Surface Disease Index; PM: particulate matter; PM_2.5_: particulate matter at size 2.5 microns and smaller; PM_10_: particulate matter at size 10 microns and smaller. ^β^: More abnormal value from each eye used in analysis.

**Table 3:** Frequency of individuals who made changes based on intervention.

Intervention	# Participants Who Implemented Intervention (%) (n)
Number of interventions	
0	29.3% (29)
1	18.2% (18)
2	19.2% (19)
3 or more	33.3% (33)
Specific Interventions	
Home ventilation (opening doors and windows)	42.4% (42)
AC air filter change every 2–3 months	36.4% (36)
Exhaust fan use	31.3% (31)
Vinegar and baking soda use for cleaning	27.3% (27)
Indoor temperature < 72 °F	23.3% (23)
Indoor plants	20.2% (20)
Air purifier(s)	9.1% (9)
Indoor humidity between 50 and 55%	5.8% (6)
Ultraviolet light in the AC unit	3.0% (3)
Carpet removal	4.0% (4)

AC: Air conditioner. #: Number.

**Table 4:** Change in DED symptoms and signs scores in those that did not perform any interventions versus those who performed ≥ 1 environmental intervention.

Variable	Change (Baseline to 6 Months) in Those That Implemented 0 Interventions	Change (Baseline to 6 Months) in Those That Implemented ≥ 1 Intervention	*p* Value
DED symptoms			
DEQ5 score	−1.69 (3.53; 29)	−0.57 (3.44; 70)	0.15
OSDI score *	−0.31 (0.93; 29)	−0.14 (0.89; 70)	0.42
Corneal sensitivity			
Average detection threshold (mL/min) *	0.14 (0.77; 27)	0.22 (0.66; 68)	0.64
Average pain threshold (mL/min) *	0.02 (0.68; 27)	0.06 (0.64; 68)	0.80
Ocular surface signs ^β^			
Tear osmolarity (mOsm/L)	0.00 (0.08; 26)	0.03 (0.07; 67)	0.08
Difference in osmolarity between eyes (mOsm/L) *	−0.26 (1.53; 26)	0.40 (1.23; 67)	0.03
Sum score of eyelid laxity *	−0.14 (0.62; 29)	−0.05 (0.50; 73)	0.47
Conjunctivochalasis *	−0.18 (0.51; 29)	0.00 (0.59; 73)	0.15
Corneal staining *	−0.25 (0.91; 29)	−0.03 (0.88; 68)	0.26
Conjunctival staining *	0.03 (0.62; 29)	−0.08 (0.57; 66)	0.43
Tear breakup time (seconds) *	0.05 (0.39; 29)	−0.03 (0.55; 68)	0.50
Schirmer wetting by tears (mm) *	0.38 (0.92; 29)	0.11 (0.47; 68)	0.06
Inferior Meibomian gland plugging *	−0.08 (0.50; 28)	0.19 (0.53; 67)	0.02
Inferior Meibomian gland dropout	−0.10 (1.23; 29)	−0.01 (1.39; 68)	0.77
Home environment			
Indoor dry bulb temperature (°C)	−0.76 (3.74; 27)	0.41 (3.51; 68)	0.16
Indoor relative humidity (%)	−9.56 (29.91; 26)	−12.77 (24.57; 67)	0.60
Indoor heat stress index (°C)	−1.53 (7.65; 26)	−0.02 (5.56; 67)	0.15
Indoor airborne PM_2.5_ concentration (µg/m^3^) *	0.08 (0.85; 20)	0.07 (1.10; 58)	0.99
Indoor airborne PM_10_ concentration (µg/m^3^) *	0.28 (1.09; 20)	0.15 (1.09; 58)	0.64

* variable log transformed; DED: dry eye disease; DEQ5: 5-Item Dry Eye Questionnaire; OSDI: Ocular Surface Disease Index; PM: particulate matter; PM_2.5_: particulate matter at size 2.5 microns and smaller; PM_10_: particulate matter at sizes 10 microns and smaller. ^β^: More abnormal value from each eye used in analysis.

**Table 5:** Impact of the number of interventions implemented during the last six months on the change in DED signs (95% confidence interval in parenthesis).

	log_e_ (Δosmolarity mOsmol/L)	log_e_ (Δosmolarity Difference Between Eyes mOsmol/L)	log_e_ (ΔMeibomian Gland Plugging)
# of interventions (0 = none, 1, 2, and 3 = 3 or more)	0.02 **	0.28 **	0.14 ***
	(0.00–0.03)	(0.03–0.54)	(0.05–0.24)
Age categories (1 = < 45 years; 2 = 45–62; 3 = 62+)	0.02	0.25	−0.02
	(−0.01–0.05)	(−0.26–0.77)	(−0.21–0.18)
Gender (1 = male; 2 = female)	−0.01	0.16	−0.08
	(−0.06–0.03)	(−0.72–1.05)	(−0.41–0.25)
Season (1 = winter and spring; 2 = summer and fall)	0	0.35	−0.1
	(−0.03–0.03)	(−0.22–0.93)	(−0.32–0.11)
Race (1 = White; 0 = other)	0	0.08	0.04
	(−0.03–0.03)	(−0.48–0.65)	(−0.18–0.25)
Allergy status (1 = yes, 0 = otherwise)	−0.02	−0.4	0.13
	(−0.05–0.01)	(−0.98–0.17)	(−0.09–0.34)
Factor 1—Comorbidities ^α1^	0.03 **	−0.02	0.07
	(0.00–0.05)	(−0.50–0.46)	(−0.11–0.25)
Factor 2—Comorbidities ^α2^	−0.03 **	−0.2	0.03
	(−0.05–−0.00)	(−0.64–0.24)	(−0.13–0.19)
Factor 1—Medicine use ^γ1^	0.02 *	0.26 *	−0.02
	(−0.00–0.03)	(−0.05–0.57)	(−0.13–0.10)
Factor 2—Medicine use ^γ2^	−0.03 **	−0.34	−0.15 *
	(−0.05–−0.00)	(−0.79–0.10)	(−0.31–0.01)
Constant	−0.02	−1.04	−0.01
	(−0.10–0.06)	(−2.59–0.51)	(−0.60–0.57)
Observations	92	92	94
R-squared	0.21	0.14	0.17

^α1^ refers to factor 1 of comorbidities, which has dominant loading of hypertension, hypercholesterolemia, arthritis, benign prostatic hyperplasia, and sleep apnea. ^α2^ refers to factor 2, which has dominant loading for hepatitis C and rosacea and negative weight for hypercholesterolemia. ^γ1^ refers to factor 1 of medicine use, which includes dominant loading for antidepressant and antianxiety medications. ^γ2^ refers to factor 2 of medicine use which has dominant loadings for aspirin, multivitamin, and medicine for cholesterol. #: Number. * = *p*-value ≤ 0.1; ** = *p*-value ≤ 0.05; *** = *p*-value ≤ 0.01.

**Table 6:** Impact of home ventilation on change in DED signs (95% confidence interval in parenthesis).

	log_e_ (Δosmolarity mOsmol/L)	log_e_ (Δosmolarity Difference Between Eyes mOsmol/L	log_e_ (ΔSchirmer Wetting mm)	log_e_ (ΔMeibomian Gland Plugging)
Home ventilation	0.03 **	0.72 **	−0.28 *	0.32 ***
	(0.00–0.07)	(0.12–1.32)	(−0.56–0.00)	(0.08–0.55)
Age categories (1 = < 45 years; 2 = 45–62; 3 = 62+)	0.02	0.21	0.27 **	−0.03
	(−0.01–0.04)	(−0.30–0.72)	(0.03–0.51)	(−0.23–0.16)
Gender (1 = male; 2 = female)	0	0.33	0.28	−0.01
	(−0.05–0.04)	(−0.54–1.20)	(−0.14–0.69)	(−0.35–0.32)
Season (1 = winter and spring; 2 = summer and fall)	−0.01	0.34	0.03	−0.11
	(−0.04–0.02)	(−0.23–0.91)	(−0.24–0.29)	(−0.33–0.11)
Race (1 = White; 0 = other)	0	0.13	−0.2	0.07
	(−0.03–0.03)	(−0.43–0.69)	(−0.46–0.06)	(−0.14–0.28)
Allergy status (1 = yes, 0 = otherwise)	−0.02	−0.32	0.02	0.17
	(−0.05–0.01)	(−0.89–0.26)	(−0.24–0.29)	(−0.05–0.38)
Factor 1—Comorbidities ^α1^	0.03 *	−0.02	−0.11	0.07
	(−0.00–0.05)	(−0.50–0.46)	(−0.33–0.11)	(−0.12–0.25)
Factor 2—Comorbidities ^α2^	−0.02 **	−0.14	−0.15	0.05
	(−0.05–−0.00)	(−0.58–0.30)	(−0.35–0.05)	(−0.11–0.21)
Factor 1—Medicine use ^γ1^	0.01	0.24	−0.01	−0.02
	(−0.00–0.03)	(−0.07–0.54)	(−0.15–0.13)	(−0.13–0.09)
Factor 2—Medicine use ^γ2^	−0.02 **	−0.31	−0.02	−0.14 *
	(−0.05–−0.00)	(−0.75–0.13)	(−0.22–0.17)	(−0.30–0.02)
Constant	−0.01	−1.04	−0.56	0
	(−0.10–0.07)	(−2.58–0.50)	(−1.28–0.17)	(−0.59–0.59)
Observations	92	92	96	94
R-squared	0.19	0.15	0.16	0.16

^α1^ refers to factor 1 of comorbidities, which has dominant loading of hypertension, hypercholesterolemia, arthritis, benign prostatic hyperplasia, and sleep apnea. ^α2^ refers to factor 2, which has dominant loading for hepatitis C and rosacea and negative weight for hypercholesterolemia. ^γ1^ refers to factor 1 of medicine use, which includes dominant loading for antidepressant and antianxiety medications. ^γ2^ refers to factor 2 of medicine use which has dominant loadings for aspirin, multivitamin, and medicine for cholesterol. * = *p*-value ≤ 0.1; ** = *p*-value ≤ 0.05; *** = *p*-value ≤ 0.01.

## Data Availability

The data presented in this study are available on request from the corresponding author due to privacy concerns.

## Supplementary Materials

The following supporting information can be downloaded at https://www.mdpi.com/article/10.3390/ijerph22030438/s1, Table S1: The use of exhaust fan and changes in DED symptoms and signs.; Table S2: The impact of using baking soda and vinegar on changes in DED signs.; Table S3: The impact of keeping temperature below 72 °F on change in DED symptoms and signs (95% confidence interval in parenthesis).; Table S4: The impact on indoor plants on changes in DED symptoms and signs (95% confidence interval in parenthesis).
