# Supplementary material for: The Impact of Home Interventions on Dry Eye Disease (DED) Symptoms and Signs in United States Veterans
Source: Int J Environ Res Public Health. 2025 Mar 17;22(3):438. doi: 10.3390/ijerph22030438 (PMC11941956; doi:10.3390/ijerph22030438)
Supplement: Supplementary file 1 [file ijerph-22-00438-s001.zip › ijerph-3329587-supplementary.pdf]

Supplement Online Material

Table S1. Use of exhaust fan and changes in DED symptoms and signs.

Variables	$\log_e$ (ΔOSDI score)	$\log_e$ (Δ osmolarity mOsmol/L)	$\log_e$ (ΔSchirmer wetting length (mm))
Exhaust fan use	0.33*	0.03*	-0.21
	(-0.06 - 0.72)	(-0.00 - 0.06)	(-0.51 - 0.08)
Age categories (1= < 45 years; 2 =45-62; 3 = 62+)	-0.01	0.02	0.26**
	(-0.32 - 0.31)	(-0.01 - 0.04)	(0.02 - 0.50)
Gender (1 = male; 2 =female)	0.27	0	0.28
	(-0.25 - 0.80)	(-0.05 - 0.05)	(-0.13 - 0.70)
Season (1=winter & spring; 2=summer 7& fall)	-0.18	-0.01	0.04
	(-0.53 - 0.18)	(-0.04 - 0.02)	(-0.23 - 0.31)
Race (1 = White; 0 = otherwise)	-0.02	0	-0.2
	(-0.36 - 0.33)	(-0.03 - 0.03)	(-0.46 - 0.06)
Allergy status (1 = yes, 0=otherwise)	-0.1	-0.02	0.04
	(-0.46 - 0.26)	(-0.05 - 0.01)	(-0.22 - 0.31)
Factor 1 – Comorbidities$^{\alpha1}$	0.21	0.02*	-0.08
	(-0.08 - 0.51)	(-0.00 - 0.05)	(-0.30 - 0.14)
Factor 2 - Comorbidities $^{\alpha2}$	0.13	-0.03**	-0.13
	(-0.14 - 0.40)	(-0.05 - -0.00)	(-0.33 - 0.07)
Factor 1 – Medicine use$^{\gamma1}$	0.11	0.01	0
	(-0.08 - 0.30)	(-0.00 - 0.03)	(-0.14 - 0.14)
Factor 2 – Medicine use $^{\gamma2}$	-0.13	-0.02**	-0.03
	(-0.39 - 0.14)	(-0.05 - -0.00)	(-0.23 - 0.16)
	-0.41	0.01	-0.61
Constant	(-1.39 - 0.57)	(-0.01 - 0.03)	(-1.34 - 0.12)
Observations	98	93	96
R-squared	0.11	0.04	0.15
(* = p-value ≤ 0.1; ** = p-value ≤ 0.05; *** = p-value ≤ 0.01)			

$^{\alpha1}$ refers to factor 1 of comorbidities, which dominant loading of hypertension, hypercholesterolemia, arthritis, benign prostatic hyperplasia, and sleep apnea.

$^{\alpha2}$ refers to factor 2, which has dominant loading for hepatitis C and rosacea and negative weight for hypercholesterolemia.

$^{\gamma1}$ refers to factor 1 of medicine use, which includes dominant loading for antidepressant and antianxiety medications.

$^{\gamma2}$ refers to o factor 2 of medicine use which has dominant loadings for aspirin, multivitamin, medicine for cholesterol.

Table S2 – Impact of using baking soda and vinegar on changes in DED signs.

Variables	$\log_e$ (Δ changes in conjunctival staining scale)	$\log_e$ (Δ meibomian gland plugging scale)
Started using vinegar and baking soda for cleaning (1=yes, 0 otherwise)	-0.27*	0.22*
	(-0.56 - 0.03)	(-0.03 - 0.48)
Age categories (1= < 45 years; 2 =45-62; 3 = 62+)	-0.03	-0.03
	(-0.26 - 0.19)	(-0.23 - 0.17)
Gender (1 = male; 2 =female)	-0.04	-0.07
	(-0.45 - 0.36)	(-0.42 - 0.27)
Season (1=winter & spring; 2=summer 7& fall)	0.19	-0.19*
	(-0.06 - 0.43)	(-0.41 - 0.03)
Race (1 = white; 0 = otherwise)	0.05	0.09
	(-0.19 - 0.29)	(-0.13 - 0.30)
Allergy status (1 = yes, 0 = otherwise)	-0.15	0.1
	(-0.40 - 0.10)	(-0.12 - 0.32)
Factor 1 – Comorbidities$^{\alpha 1}$	-0.11	0.02
	(-0.31 - 0.09)	(-0.16 - 0.20)
Factor 2 - Comorbidities $^{\alpha 2}$	-0.14	0.02
	(-0.32 - 0.05)	(-0.14 - 0.19)
Factor 1 – Medicine use$^{\gamma 1}$	-0.03	-0.01
	(-0.16 - 0.11)	(-0.13 - 0.11)
Factor 2 – Medicine use $^{\gamma 2}$	0.07	-0.11
	(-0.11 - 0.25)	(-0.27 - 0.05)
Constant	0.1	0.2
	(-0.57 - 0.77)	(-0.39 - 0.80)
Observations	94	94
R-squared	0.12	0.11
* = p-value ≤ 0.1; ** = p-value ≤ 0.05; *** = p-value ≤ 0.01		

$^{\alpha 1}$ refers to factor 1 of comorbidities, which dominant loading of hypertension, hypercholesterolemia, arthritis, benign prostatic hyperplasia, and sleep apnea.
$^{\alpha 2}$ refers to factor 2, which has dominant loading for hepatitis C and rosacea and negative weight for hypercholesterolemia.
$^{\gamma 1}$ refers to factor 1 of medicine use, which includes dominant loading for antidepressant and antianxiety medications.
$^{\gamma 2}$ refers to o factor 2 of medicine use which has dominant loadings for aspirin, multivitamin, medicine for cholesterol.

Table S3: Impact of keeping temperature below 72° F on change in DED symptoms and signs (95% confidence interval in parenthesis).

	Δ DEQ5 score	log_e (Δ changes in corneal staining scale)	log_e (Δ Meibomian gland plugging scale)	ΔMeibomian gland dropout
Air purifier use	-2.15**	-0.43*	0.2	-0.79**
	(-3.89 - -0.41)	(-0.87 - 0.01)	(-0.07 - 0.47)	(-1.49 - -0.08)
Age categories (1= < 45 years; 2 =45-62; 3 = 62+)	0.84	0.17	-0.02	-0.3
	(-0.42 - 2.10)	(-0.15 - 0.49)	(-0.23 - 0.18)	(-0.81 - 0.22)
Gender (1 = male; 2 =female)	-0.69	0	0	-0.16
	(-2.78 - 1.40)	(-0.56 - 0.55)	(-0.35 - 0.34)	(-1.05 - 0.72)
Season (1=winter & spring; 2=summer 7& fall)	-0.75	-0.23	-0.14	-0.17
	(-2.18 - 0.67)	(-0.59 - 0.13)	(-0.37 - 0.08)	(-0.75 - 0.40)
Race (1 = White; 0 = otherwise)	-0.84	0.14	0.07	0.15
	(-2.24 - 0.57)	(-0.22 - 0.49)	(-0.15 - 0.29)	(-0.42 - 0.71)
Allergy status (1 = yes, 0=otherwise)	0.3	-0.3	0.13	-0.29
	(-1.14 - 1.73)	(-0.65 - 0.06)	(-0.10 - 0.35)	(-0.86 - 0.28)
Factor 1 – Comorbidities$^{\alpha 1}$	0.26	-0.21	0.01	0.05
	(-0.90 - 1.43)	(-0.50 - 0.08)	(-0.17 - 0.19)	(-0.41 - 0.52)
Factor 2 - Comorbidities $^{\alpha 2}$	-0.53	0.14	0.03	0.1
	(-1.62 - 0.55)	(-0.13 - 0.41)	(-0.14 - 0.20)	(-0.33 - 0.53)
Factor 1 – Medicine use$^{\gamma 1}$	-0.1	-0.16*	-0.02	-0.04
	(-0.85 - 0.64)	(-0.35 - 0.03)	(-0.14 - 0.09)	(-0.34 - 0.26)
Factor 2 – Medicine use $^{\gamma 2}$	-0.18	-0.03	-0.11	-0.04
	(-1.24 - 0.87)	(-0.30 - 0.23)	(-0.27 - 0.05)	(-0.46 - 0.38)
Constant	-0.92	-0.18	0.1	1.19
	(-4.82 - 2.99)	(-1.15 - 0.79)	(-0.51 - 0.70)	(-0.37 - 2.75)
Observations	98	96	94	96
R-squared	0.14	0.17	0.11	0.09
* = p-value ≤ 0.1; ** = p-value ≤ 0.05; *** = p-value ≤ 0.01				

$^{\alpha 1}$ refers to factor 1 of comorbidities, which dominant loading of hypertension, hypercholesterolemia, arthritis, benign prostatic hyperplasia, and sleep apnea.

$^{\alpha 2}$ refers to factor 2, which has dominant loading for hepatitis C and rosacea and negative weight for hypercholesterolemia.

$^{\gamma 1}$ refers to factor 1 of medicine use, which includes dominant loading for antidepressant and antianxiety medications.

$^{\gamma 2}$ refers to o factor 2 of medicine use which has dominant loadings for aspirin, multivitamin, medicine for cholesterol.

Table S4: Impact on indoor plants on changes in DED symptoms and signs (95% confidence interval in parenthesis).

	log_e (ΔOSDI score)	log_e (ΔMeibomian gland plugging scale)
Air purifier use	0.52**	0.28**
	(0.08 - 0.96)	(0.00 - 0.56)
Age categories (1= < 45 years; 2 =45-62; 3 = 62+)	-0.04	-0.06
	(-0.36 - 0.27)	(-0.26 - 0.14)
Gender (1 = male; 2 =female)	0.21	-0.02
	(-0.30 - 0.73)	(-0.36 - 0.32)
Season (1=winter & spring; 2=summer 7& fall)	-0.23	-0.17
	(-0.58 - 0.11)	(-0.38 - 0.05)
Race (1 = White; 0 = otherwise)	0.03	0.1
	(-0.31 - 0.38)	(-0.11 - 0.32)
Allergy status (1 = yes, 0=otherwise)	-0.18	0.09
	(-0.54 - 0.17)	(-0.13 - 0.32)
Factor 1 – Comorbidities$^{\alpha 1}$	0.22	0.03
	(-0.07 - 0.51)	(-0.15 - 0.21)
Factor 2 - Comorbidities $^{\alpha 2}$	0.15	0.04
	(-0.11 - 0.42)	(-0.13 - 0.20)
Factor 1 – Medicine use$^{\gamma 1}$	0.14	-0.02
	(-0.04 - 0.32)	(-0.14 - 0.10)
Factor 2 – Medicine use $^{\gamma 2}$	-0.12	-0.12
	(-0.38 - 0.14)	(-0.28 - 0.04)
Constant	-0.21	0.21
	(-1.16 - 0.73)	(-0.38 - 0.79)
Observations	98	94
R-squared	0.13	0.13
* = p-value ≤ 0.1; ** = p-value ≤ 0.05; *** = p-value ≤ 0.01		

$^{\alpha 1}$ refers to factor 1 of comorbidities, which dominant loading of hypertension, hypercholesterolemia, arthritis, benign prostatic hyperplasia, and sleep apnea.

$^{\alpha 2}$ refers to factor 2, which has dominant loading for hepatitis C and rosacea and negative weight for hypercholesterolemia.

$^{\gamma 1}$ refers to factor 1 of medicine use, which includes dominant loading for antidepressant and antianxiety medications.

$^{\gamma 2}$ refers to o factor 2 of medicine use which has dominant loadings for aspirin, multivitamin, medicine for cholesterol.
